# Patterns of genetic variation in the endangered European mink (*Mustela lutreola* L., 1761)

**DOI:** 10.1186/s12862-015-0427-9

**Published:** 2015-07-17

**Authors:** Maria Teresa Cabria, Elena G. Gonzalez, Benjamin J. Gomez-Moliner, Johan R. Michaux, Dimitry Skumatov, Andreas Kranz, Pascal Fournier, Santiago Palazon, Rafael Zardoya

**Affiliations:** Departamento de Zoología y B.C.A., Facultad de Farmacia, Universidad del País Vasco UPV/EHU, Paseo de las Universidades, 7, 01006 Vitoria-Gasteiz, Spains; Laboratoire de Biologie Évolutive, Institut de Botanique (Bat.22), Université de Liège (Sart Tilman), Boulevard du Rectorat, 27, B4000 Liège, Belgium; Departamento de Biodiversidad y Biología Evolutiva, Museo Nacional de Ciencias Naturales, CSIC, José Gutiérrez Abascal, 2, 28006 Madrid, Spain; Centre de Biologie et de Gestion des Populations, CBGP, Campus international de Baillarguet, CS 30016, 34988 Montferrier-sur-Lez, Cedex France; Russian Research Institute of Game Management and Fur Farming, Engels Street, 79, Kirov, Russia; Institute of Wildlife Biology and Game Management, BOKU University of Natural Resources and Life Sciences, Gregor Mendel Street 33, 1180 Vienna, Austria; Groupe de Recherche et d’Etudes pour la Gestion de l’Environnement (GREGE), Route de Préchac, 33730 Villandraut, France; Departament de Biologia Animal, Facultat de Biologia, Universitat de Barcelona, Diagonal 643, 2ª planta, 08028 Barcelona, Spain

## Abstract

**Background:**

The European mink (*Mustela lutreola*, L. 1761) is a critically endangered mustelid, which inhabits several main river drainages in Europe. Here, we assess the genetic variation of existing populations of this species, including new sampling sites and additional molecular markers (newly developed microsatellite loci specific to European mink) as compared to previous studies. Probabilistic analyses were used to examine genetic structure within and between existing populations, and to infer phylogeographic processes and past demography.

**Results:**

According to both mitochondrial and nuclear microsatellite markers, Northeastern (Russia, Estonia and Belarus) and Southeastern (Romania) European populations showed the highest intraspecific diversity. In contrast, Western European (France and Spain) populations were the least polymorphic, featuring a unique mitochondrial DNA haplotype. The high differentiation values detected between Eastern and Western European populations could be the result of genetic drift in the latter due to population isolation and reduction. Genetic differences among populations were further supported by Bayesian clustering and two main groups were confirmed (Eastern vs. Western Europe) along with two contained subgroups at a more local scale (Northeastern vs. Southeastern Europe; France vs. Spain).

**Conclusions:**

Genetic data and performed analyses support a historical scenario of stable European mink populations, not affected by Quaternary climate oscillations in the Late Pleistocene, and posterior expansion events following river connections in both North- and Southeastern European populations. This suggests an eastern refuge during glacial maxima (as already proposed for boreal and continental species). In contrast, Western Europe was colonised more recently following either natural expansions or putative human introductions. Low levels of genetic diversity observed within each studied population suggest recent bottleneck events and stress the urgent need for conservation measures to counteract the demographic decline experienced by the European mink.

**Electronic supplementary material:**

The online version of this article (doi:10.1186/s12862-015-0427-9) contains supplementary material, which is available to authorized users.

## Background

The European mink (*Mustela lutreola*, L. 1761) is a riparian mustelid that used to occupy most of the main river drainages in Europe. The rapid decline of this species throughout its distribution range [1, 2] is the result of its dependence on natural river courses that limits its dispersal capacity [3, 4], and factors such as habitat fragmentation, over-hunting, pollution, anthropogenic barriers, as well as the presence of the invasive American mink (*Neovison vison*), a direct ecological competitor potentially spreading diseases to which *M. lutreola* is vulnerable [5–8]. Consequently, the European mink is listed as one of the most endangered mammals in Europe (IUCN Red List of Threatened Species, http://www.iucnredlist.org) [9]. In an effort to promote its long-term conservation, several measures are under consideration (i.e., captive breeding, restoration and/or reintroduction programs). These measures seek to maintain the genetic diversity of wild populations of this endangered species.

At present, the European mink consists of scattered isolated populations restricted to three areas: Northeastern Europe (NE; Belarus and Russia), Southeastern Europe (SE; Danube and Dniester deltas in Romania and Ukraine), and Western Europe (W; Southwest France and Northern Spain) [6, 9]. To date, genetic studies on the European mink [10, 11] have examined mitochondrial (mt) DNA control region, and six microsatellite loci originally isolated from other mustelid species. These studies pointed to NE Europe as the most likely glacial refuge since the population in this region showed the highest haplotype diversity. In addition, the extremely low genetic diversity of the W population was interpreted as evidence for a recent colonization of this region by few animals, possibly after human introduction [10]. However, the specimens examined in these studies [10, 11] were mostly from France and results require further confirmation based on a more complete and even sampling effort across the current distribution range of the species.

In the present study, we combined molecular data (both mtDNA and nuclear microsatellites) from Michaux *et al.* [10] with new molecular data obtained from additional specimens from Russia, Belarus, Romania, and Spain. We also used five new species-specific microsatellite loci isolated from European mink [12] on all samples. With these new data and recently developed Bayesian analysis methods, our goals were to gain further insight into: i) the current genetic structure of the European mink; ii) the phylogeographic processes associated with the glacial refuge and post-glacial colonization of the species, and the origin of the W population; iii) the role of river drainages in shaping the current species distribution; and iv) the existence of population genetic bottlenecks caused by the effects of human impact. By improving our understanding of the evolutionary processes leading to the current population structure of the European mink, the results of this study are useful to shape future management strategies for the conservation of this endangered species.

## Results

### Mitochondrial DNA analyses

#### Genetic variability-Standard population genetic analyses

**Fig. 1:**
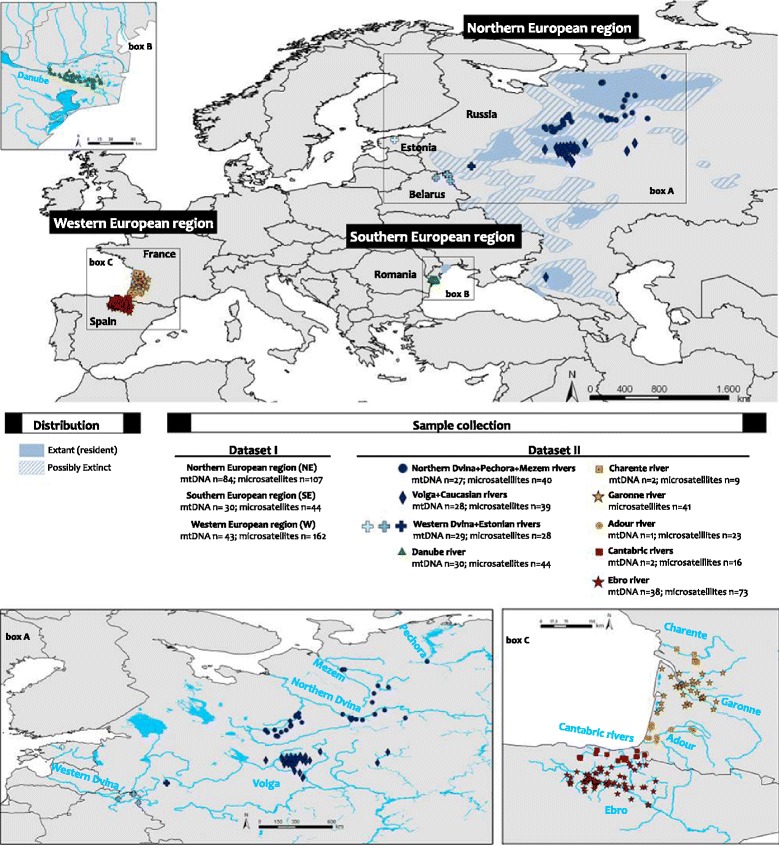
Geographical map depicting the distribution area (according to the IUCN), and sample collection of *Mustela lutreola.* The distribution area shows where the species currently lives (*shaded area*) and is possibly extinct (*hatched area*). Sampling sites are indicated by colours (Russia dark blue; Belarus blue; Estonia light blue; Romania green; France orange; Spain red) and drainage basins by shape (circle; diamond; cross; triangle; square; star). The numbers of samples analysed for microsatellite markers and mtDNA are also indicated

A fragment of the mtDNA control region was amplified and sequenced in 157 specimens (Fig. 1). The trimmed alignment (after removing indels) was 476-bp long and yielded 17 distinct haplotypes defined by 17 variable sites. Sequence variability of mtDNA was characterised by nucleotide and haplotype diversities of π = 0.005 ± 0.003 and *h* = 0.857 ± 0.014, respectively (Table 1). The mtDNA dataset I revealed highest levels of genetic diversity (π = 0.004 and *h* = 0.862) and a high percentage (92.31 %) of private haplotypes for NE populations (mostly Russia). In contrast, SE populations were characterised by four haplotypes (of which, only Mlh16 was shared with Russia) and low mtDNA variation (π = 0.0019 and *h* = 0.352). Lowest haplotype diversity was detected for W populations, where all individuals shared the same haplotype, which was not found in Eastern European (NE and SE) populations. Analyses based on the mtDNA dataset II identified rivers North Dvina, West Dvina and Volga (NE populations) as significantly contributing to mean genetic diversity levels (Table 1).

**Table 1:** MtDNA diversity estimates and neutrality test results for European mink dataset I^a^ and dataset II^b^. The variables provided are: number of sampled individuals (n), number of observed haplotypes (N_*h*_) with private haplotypes (P_*h*_) in brackets, and haplotype (*h*) and nucleotide (*π*) diversities with standard deviations (SD) in brackets. None of the neutrality tests performed were significant (*P* > 0.05)

			Diversity indices		Neutrality test		
Sampling sites	n	N_*h*_ (P_*h*_)	*h* (SD)	*π* (SD)	Tajima’s *D*	Fu's *Fs*	*R2*
All individuals tested	157	17	0.857 (0.014)	0.005 (0.003)	−0.399	−3.316	0.075
mtDNA dataset I							
East (Russia, Belarus, Estonia, Romania)	114	16(16)	0.869 (0.014)	0.005 (0.003)	−0.550	−3.380	0.075
Northeast (Russia, Belarus, Estonia)	84	13 (12)	0.862 (0.016)	0.004 (0.003)	−1.008	−3.501	0.067
Southeast (Romania)	30	4 (3)	0.352 (0.103)	0.0019 (0.0015)	−0.890	0.012	0.087
mtDNA dataset II							
North Dvina	27	6	0.775 (0.047)	0.004 (0.002)	0.817	−0.287	0.165
Volga	28	4	0.717 (0.050)	0.003 (0.002)	0.515	0.865	0.154
West Dvina	29	10	0.810 (0.064)	0.005 (0.003)	−1.117	−2.177	0.086

^a^Because of the presence of only a single haplotype in the West, this region is not included in the table

^b^Results for the Danube river correspond to those obtained for the southeastern region

#### Phylogenetic analyses and population genetic structure

**Fig. 2:**
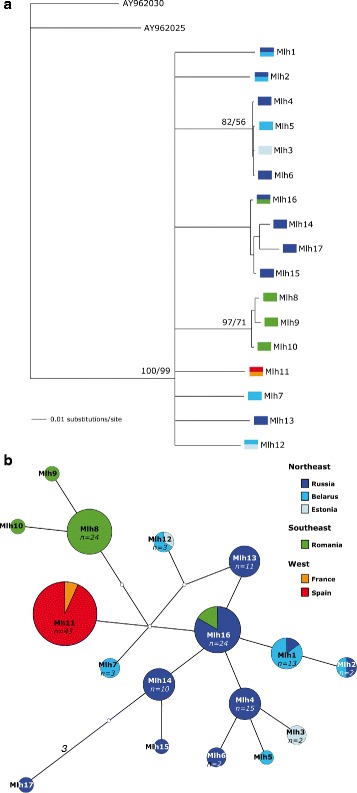
Phylogenetic tree and median-joining network. (**a**) Phylogenetic relationships of the European mink based on mtDNA haplotype data using BI and ML. Numbers represent support for BI (BPPs, on the left) and ML (BPs, on the right). (**b**) Median-joining network showing the relationships among mtDNA haplotypes of *Mustela lutreola* (ε = 10). Circle sizes are proportional to the haplotype frequency. White circles show the missing intermediate haplotype states, and connections represent one mutation step (except for the branch indicated in italics). The geographical origin of each haplotype is indicated by different colours (see legend)

The reconstructed phylogeny showed a general lack of resolution. The only strongly supported clade included haplotypes from the SE populations (Fig. 2a). The haplotype network (Fig. 2b) revealed that most NE haplotypes are connected to each other (generally differing by only one mutational step) in a star-like fashion with a central haplotype (Mlh16), which is also found in SE populations. SE and W populations showed private haplotypes, which are connected to the network through missing intermediate haplotypes. Reconstruction of the haplotype network using statistical parsimony rendered similar results (data not shown).

High levels of haplotype frequency differentiation (Φ_ST_ values ranged from 0.586 to 0.879) were found among NE, SE, and W populations (mtDNA dataset I), with all comparisons being statistically significant (Additional file 1). A more detailed analysis comparing the different sampling localities also showed significant Φ_ST_ values, ranging from 0.143 (Russia vs. Belarus + Estonia) to 0.742 (Belarus + Estonia vs. W) (data not shown). Similarly, significant genetic differentiation was also detected among the tested river basins (mtDNA dataset II; Additional file 1).

The hierarchical analysis of molecular variance (AMOVA) strongly supported genetic differentiation of the European mink, but failed to detect geographic structuring of East (NE + SE) vs. W populations or NE vs. SE populations (Additional file 2). The genetic variability was mainly explained by variation among populations within each region. Similarly, no evidence of genetic structuring was observed among regions when pooling individuals according to the different drainage basins (Additional file 2).

#### Historical demography

Tajima’s D, Fu’s Fs and R2 statistic tests failed to reject the null hypotheses (Ho) of non-expanding (stable) populations, as was shown by the general pattern of non-significant (*P* > 0.10) low negative Fs and Tajima’s D values (Table 1). However, most D and Fs values were negative indicating an excess of rare variants and suggesting that the overall population size may have fluctuated in the past.

**Fig. 3:**
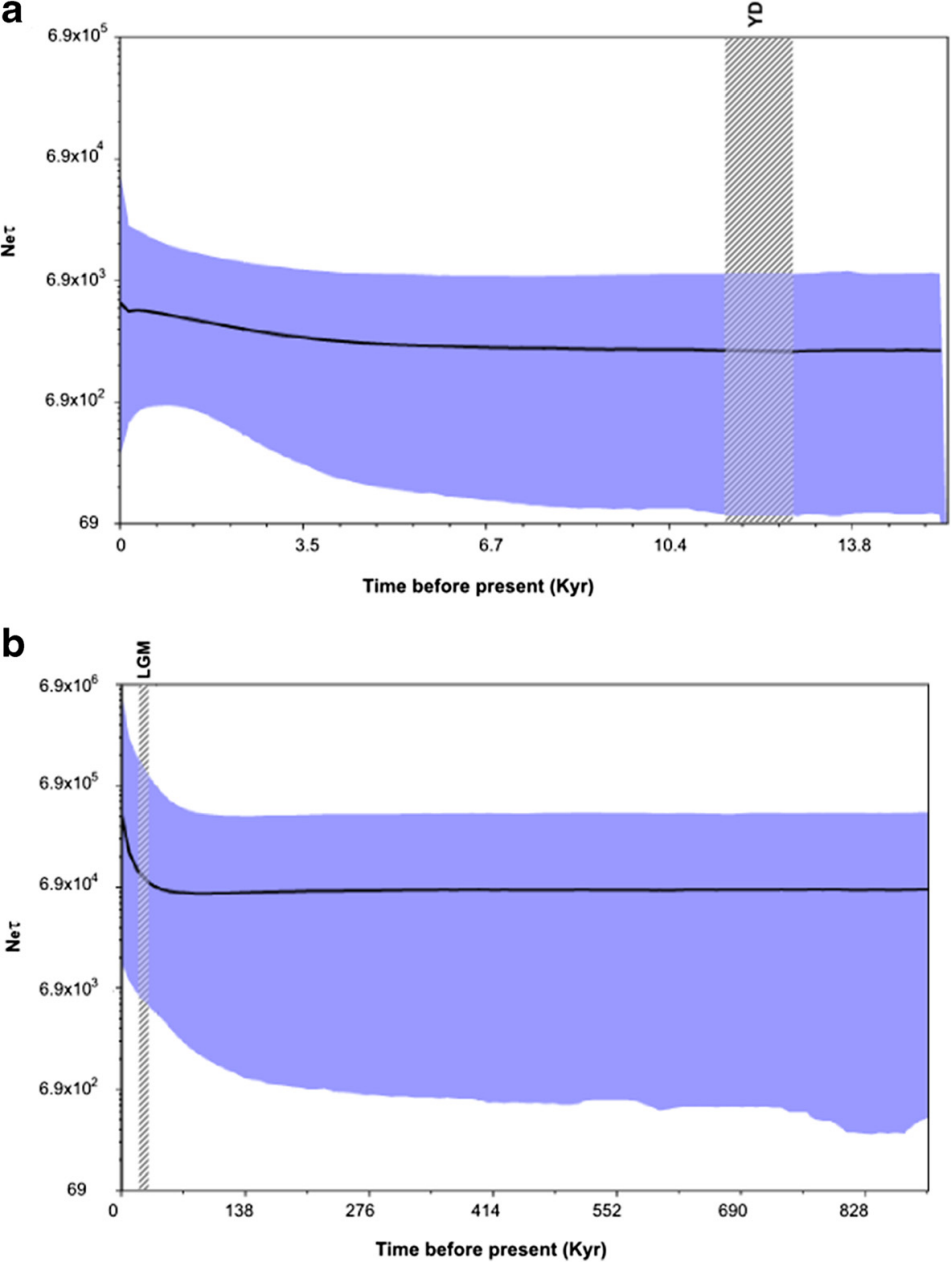
Bayesian skyline plot. Bayesian skyline plot depicting the historical demography of the Northeastern (**a**) and Southeastern (**b**) European populations of *Mustela lutreola* based on mt haplotype data. The y-axis is equal to N_e_ τ, where Ne represents the effective population size and τ the generation time in years. The x-axis represents units of time before the present in thousands of years (Kyr). The thick solid line represents the median estimated Ne, and the coloured area shows the 95 % HPD limits of N_e_ τ. The Last Glacial Maximum (LGM, 22–18 Kyr) and Younger Dryas interstadial (YD, 12.8–11.5 Kyr) are also indicated

Coalescent-based reconstructions of the demographic history of the NE and SE populations inferred from BSP analyses revealed histories of long-term stability in effective population size until approximately 5 Kyr for the NE populations, and a recent demographic expansion period in the SE populations during the Last Glacial Maximum, corresponding to a population increase of more than 30 % (Fig. 3).

### Microsatellite results

#### Genetic variability-Standard population genetic analyses

Microsatellite alleles were obtained from a total of 313 specimens (Fig. 1). Estimates of null allele frequencies were moderate to low (<0.2), and F_ST_ values obtained from the null-allele corrected and original data sets were very similar (data not shown). Thus all loci were used in further analyses.

No evidence of linkage disequilibrium was found as none of the corresponding exact tests remained significant after Bonferroni correction (*P* = 0.000182; *n* = 275 comparisons). Thus, microsatellite loci were considered statistically independent.

Microsatellites were polymorphic in all tested populations with the exception of locus MLUT04, which presented a single allele within the Spanish samples. A total of 64 alleles (ranging from two to 10 alleles per population) were obtained. Private alleles were found in both Eastern (NE + SE) and W populations but at different percentages, 52.46 % and 9.38 %, respectively (Table 2). Low levels of microsatellite genetic variability (A: 5.818; H_O_ = 0.430; H_E_ = 0.578) were detected. After Bonferroni correction, only the Spanish population showed significant departures (*P* < 0.001, *n* = 55 comparisons) from HWE for the following loci: MVIS22 (F_IS_ = 0.265), MVIS72 (F_IS_ = 0.274), and MER41 (F_IS_ = 0.422), due to a heterozygote deficit.

**Table 2:** Genetic variability estimates for eleven microsatellite loci tested in the European mink datasets I^a^ and II^b^. The variable provided are: number of individuals tested (n), number of total alleles (N_A_), the total private allele (P_A_) with the corresponding percentage in brackets, allelic diversity (A), observed and expected heterozygosities, H_O_ and H_E_ respectively, and mean F_IS_ (Wright’s statistic)

Sampling sites	n	N_A_	P_A_ (%)	A	H_O_	H_E_	F_IS_
All individuals tested	313	64	—	5.818	0.430 ± 0.113	0.578 ± 0.148	0.255
Microsatellite dataset I							
East (North and South)	151	61	32 (52.46 %)	5.546	0.532 ± 0.150	0.618 ± 0.156	0.141
Northeast	107	59	20 (33.90 %)	5.364	0.559 ± 0.153	0.613 ± 0.164	0.089
Russia	88	57	13 (22.81 %)	5.182	0.569 ± 0.151	0.619 ± 0.159	0.082
Belarus + Estonia	19	42	2 (4.76 %)	3.818	0.503 ± 0.230	0.54 ± 0.207	0.095
Southeast(Romania)	44	35	2 (5.71 %)	3.182	0.464 ± 0.170	0.496 ± 0.139	0.065
West	162	32	3 (9.38 %)	2.909	0.336 ± 0.161	0.439 ± 0.201	0.236
France	73	29	1 (3.45 %)	2.636	0.389 ± 0.182	0.430 ± 0.206	0.095
Spain	89	29	1 (3.45 %)	2.636	0.291 ± 0.184	0.353 ± 0.215	0.178
Microsatellite dataset II							
North Dvina	40	54	3 (5.56 %)	4.909	0.563 ± 0.188	0.618 ± 0.181	0.090
West Dvina	28	47	2 (4.26 %)	4.273	0.546 ± 0.187	0.582 ± 0.185	0.064
Volga	39	51	2 (3.92 %)	4.636	0.560 ± 0.147	0.598 ± 0.146	0.065
Charentes	9	25	—	2.273	0.364 ± 0.223	0.409 ± 0.213	0.117
Garonne	44	33	1 (3.03 %)	3	0.373 ± 0.186	0.426 ± 0.205	0.128
Adour	23	26	—	2.364	0.451 ± 0.233	0.432 ± 0.198	−0.045
Cantabrian rivers	16	25	—	2.273	0.317 ± 0.193	0.382 ± 0.252	0.176
Ebro	73	29	—	2.636	0.285 ± 0.185	0.337 ± 0.210	0.155

^a^Because of the low number of samples from Estonia, this locality was analyzed in combination with individuals from Belarus

^b^Results for the Danube river correspond to those obtained for the southeastern region

Consistent with mtDNA results, W and NE populations (mostly Russian) had the lowest and highest levels of genetic variability, respectively, whereas SE populations showed intermediate values (Table 2). Individuals pooled according to their drainage distribution (microsatellite dataset II) showed the highest genetic variability in the Volga, North and West Dvina rivers. The individuals from the Danube showed intermediate genetic variability and within the French and Spanish rivers, the Adour and the Ebro showed the highest and lowest genetic variability values, respectively.

#### Patterns of population genetic structure

**Fig. 4:**
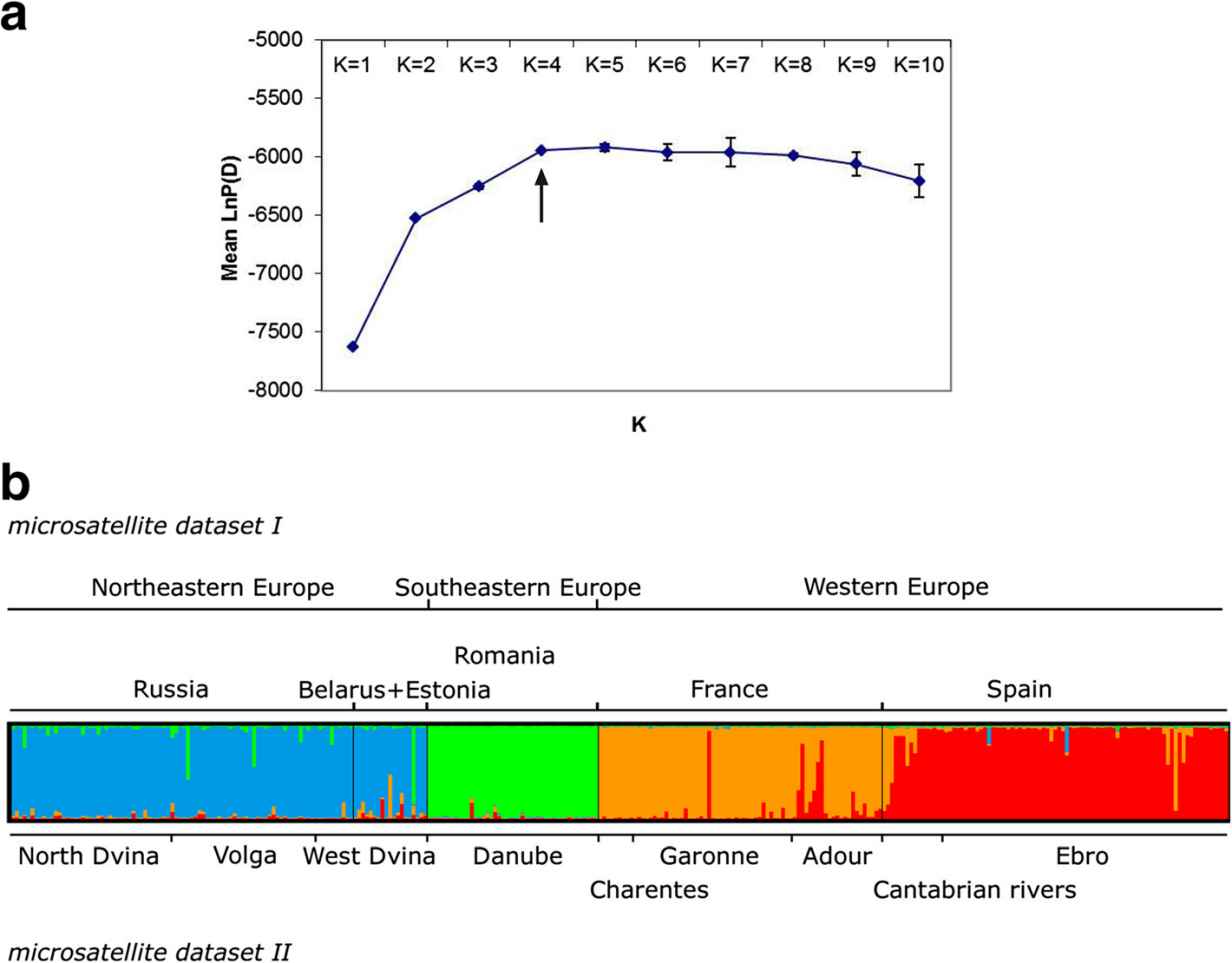
Summary of the clustering results for European mink populations obtained using STRUCTURE. (**a**) Detecting the number of populations as a function of the highest posterior probability (mean LnP[D]) over the number of clusters *k* (*k* = 4). (**b**) Each individual is represented as a vertical line partitioned into *k* segments whose length is related to their membership proportions to each inferred *k* cluster. The subdivision according to sampling localities (dataset I) or drainage distribution (dataset II) is also specified in the figure

The Bayesian clustering procedure [13] obtained the highest likelihoods for *k* = 4 (Fig. 4a). Individuals from the NE (Russia, Belarus + Estonia) populations clustered as only one inferred group with an assignment probability (Q) higher than 0.93 (Fig. 4b). Most individuals from Romania, France, and Spain were significantly assigned to three different inferred clusters (Q > 0.90). In contrast, the uppermost hierarchical level of structure was shown at *k* = 2 when the modal value of ∆k was estimated [14] (data not shown). In this case, the inferred clusters clearly corresponded to the two main regions (NE + SE versus W) analyzed, each of which showed a very high average proportion of membership (Q > 0.95) (not shown). The same results were obtained when a Dirichlet prior process was assumed [15]. The SAMOVA analysis showed the highest F_CT_ value (F_CT_ = 0.904, *P* = 0.015) for an arrangement of populations in *k* = 4 clusters, corresponding to the lineages identified by STRUCTURE.

Consistent with the mtDNA analysis, all but one pairwise F_ST_ comparisons based on microsatellite data showed significant values both at the region (microsatellite dataset I) and drainage (microsatellite dataset II) levels. The only exception was the comparison between Garonne and Charentes rivers in France (F_ST_ = 0.001; *P* < 0.0014), whose populations are geographically close (Additional file 3). Similar patterns were obtained with the R_ST_ estimates (Additional file 3). In this case, all comparisons were significant except those involving Garonne and Charentes (R_ST_ = 0.005; *P* > 0.0014), and the North Dvina and Volga (R_ST_ = 0.004; *P* > 0.0014) rivers in France and NE, respectively (Additional file 3).

The hierarchical AMOVA identified significant geographic structuring (F_ST_ = 0.224, *P* < 0.001). However, no genetic structuring was supported when STRUCTURE results (two or four geographical groups) were considered. The genetic variation in the data set was significantly explained by variation among and within populations (Additional file 4). Interestingly, genetic structuring was significant when samples were grouped according to drainage basins (Additional file 4). In agreement with other genetic analyses, FCA assigned the European mink samples to three well-defined groups, W, NE, and SE, respectively (Additional file 5).

#### Reconstruction of colonization pathways for western populations

**Fig. 5:**
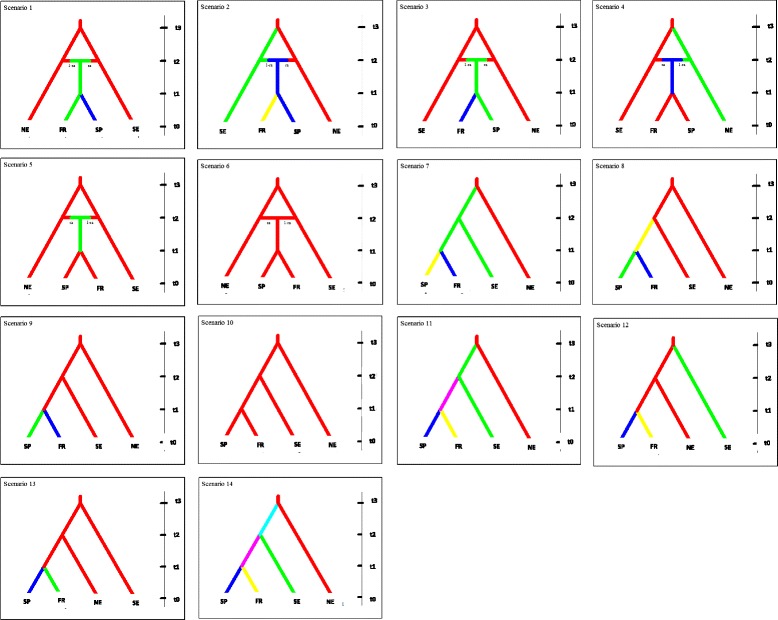
Diagram of the stepwise procedure followed for approximate Bayesian computations performed using the DIYABC program. All colonization scenarios tested assumed four current populations (NE, SE, France and Spain) and their divergence from a single ancient population. The events (size variation, divergence and/or admixture) that defined each scenario are depicted as changes in the pattern (colour or shape) in the branches of each coalescent tree. Time of events (T_i_) is shown on the right. Time 0 is the sampling time of populations. The best scenario identified in each step is also highlighted (Scenario 2, PP = 0.40)

The analysis performed under the ABC framework summarized by the polychotomous logistic regression method (Additional file 6) revealed that scenario number 2 had a significantly higher posterior probability (PP) value (0.40) as compared to other scenarios (Fig. 5).

Scenario 2 suggests a recent origin for W populations after admixture between already genetically differentiated NE and SE populations. This foundation event of the W population would have been followed by a posterior separation between French (FR) and Spanish (SP) populations in relatively recent times. The analyses gave estimations of divergence times among these populations (Additional file 7). However, these values are likely highly overestimated due to the large differences in allelic frequencies detected between the W populations associated with recent bottleneck and founder events. Therefore, we preferred to be very careful considering these estimations and not considered them as strict values in our discussion.

## Discussion

The European mink (*Mustela lutreola*, L. 1761) is one of the most endangered mammals on the continent showing only a few scattered populations restricted to three separated regions. Human pressure (e.g., habitat fragmentation and hunting) is likely the main underlying cause of the current critical situation of European mink populations. Other factors affecting population viability such as past demographic events and the ecological dependence of the species on riparian habitats might also be responsible for local declines. The present study investigates the population genetics of the European mink based on a better survey of the distribution range of *Mustela lutreola* and a more representative number of specimens per locality with respect to prior studies [10, 11]. The different analyses performed on both mt and nuclear genetic data confirmed that the overall genetic variability was relatively high, as previously reported by Michaux *et al*. [10, 11], and comparable to the levels reported for other threatened and non-threatened mustelid species [16–23]. However, our results based on both geographic (Dataset I) and river drainage (Dataset II) sampling, indicate that this genetic variation is not homogeneously distributed throughout the species’ range. Thus, Eastern European localities (NE + SE) and corresponding drainage basins (North and West Dvina, Volga and Danube rivers) revealed higher levels of genetic diversity than W localities and corresponding rivers.

### Relative contribution of historical and contemporary events to the genetic structure of the European mink

At a large geographical scale, Bayesian clustering, F_ST_ pairwise comparisons and AMOVA analyses all rejected the null hypothesis of panmixia, supporting the existence of different groups of genetic diversity among W, NE, and SE European regions. Our Bayesian clustering analysis suggests the existence of at least two main genetic units of European mink defined by the eastern (NE + SE) and W European populations, respectively. At a more local scale, Eastern Europe was differentiated into NE and SE populations, and Western Europe into French and Spanish populations. The FCA plotting analysis also supported this pattern. Isolation due to large habitat fragmentation produces strong genetic drift among regions, and this may ultimately result in the significant genetic differences (NE vs. SE vs. France vs. Spain) detected in this study.

However, our Bayesian (ABC) results also indicate that the existence of these genetic units is relatively recent, and that they were historically connected after the last glaciation. In the network reconstructions, Eastern European (NE + SE) populations retained the most abundant missing intermediate haplotypes and showed the highest levels of genetic diversity, supporting the hypothesis that the European mink survived glacial periods across an extensive geographic area covering Eastern Europe around the central Russian Upland [10, 11] in additional refugia [24] to those postulated for temperate species in the Mediterranean region [25]. Several authors suggested the Eastern Palearctic around Siberia and Southern Ural Mountains as well as possible areas of central Europe as glacial refuges for the survival of different species during cold stages of the Late Pleistocene [24, 26, 27]. The species ascribed to these refuges (e.g., *Picea abies* [28], *Rana arvalis* [29] or *Ips typographus* [30]) have in common their continental or boreal distribution. Subfossil remains of European mink dated as Upper Würmian and Holocene have been cited for the Moscow region, Estonia, Latvia Poland, Ukraine and Romania [1, 31], supporting past wide presence of the species in eastern regions. Moreover, open landscapes of tundra and steppe forests of the Late Valdai (Würm) Glaciation in the Central Russian Upland region are also in favor of such refuge hypotheses, as these habitats are particularly adequate for the survival of European mink [32, 33]. The possibility that *M. lutreola* was able to survive glacial maxima in Eastern Europe is further supported by the non-significant results of performed neutrality tests. Bayesian skyline plot analysis also indicated a scenario of stable populations, suggesting that the demographic course of European mink was not seriously affected by Quaternary climate oscillations in the Late Pleistocene.

The AMOVA results showing no significant geographic structuring among regions strengthen the hypothesis of a rapid post-glacial expansion. According to the refuge theory [25, 34], rapid population expansion from refugia during interglacial periods would have entailed serial bottlenecking with progressive loss of allelic diversity, resulting in less population genetic diversity among the most recently colonised places. The star-like pattern for the European mink NE population inferred here is consistent with this theory. However, the patterns displayed by SE and W populations depart from expectations and additional events are needed to explain these patterns (see below).

The current distribution of European mink is closely linked to river courses, which could be the main force driving the historical and current genetic structuring of the species. We tested such hypothesis, and significant differentiation was detected associated to river drainages. However, this result could be an artifact since inclusion of populations with low numbers of individuals such as the Danube River population may have lead to overestimation in the detection of genetic structure of the species. In fact, AMOVA results on samples clustered according to different rivers were non significant. This suggests that European mink may be capable of dispersal across river drainages over long periods of time, provided that natural conditions are favorable, which still seems to be the case in some parts of the NE region, and likely was throughout Europe during the rapid postglacial expansion of European mink. In particular, field studies corroborate important movement capacity of the European mink during the reproductive period in populations with low densities, when animals are looking for potential mates, moving up to 40–50 km during these periods [35].

At present, however, natural conditions in riverine habitats are not that favorable for survival in much of the distribution of the species, and the significant levels of heterozygosity excess detected together with the results of genetic bottleneck analyses suggest that European mink has suffered recent (human-mediated) genetic bottleneck processes, which might result in local extinctions. This would be consistent with the reported overall demographic decline of the species during the past century [2]. Only individuals from rivers Garonne and Ebro, as well as Belarus locality, seemed to be exceptions to the general trend of genetic depletion detected for the species. Habitat fragmentation affecting dispersal across river drainages seems to be the major force behind interrupted gene flow among populations.

The origin of the W population of European mink has been the subject of a hot debate [36–38]. Our ABC analysis favored a scenario in which Eastern populations had successive cycles of genetic differentiation (e.g., due to isolation by distance) and posterior genetic admixture (e.g., through spread across river connections). The W population would have diverged from an ancestral eastern population encompassing both NE and SE genetic pools during a period of population admixture. The alternative scenario of no genetic differentiation/ admixture cycles within the Eastern region and thus a stable and continuous distribution range of European mink over time in this region was less preferred. Afterwards, the FR and SP would have separated at both sides of the Pyrenees leading to the currently observed genetic differentiation within the W population.

The ultimate causes of the observed genetic uniformity of the W population could be explained by several working hypotheses. If European mink was not present in the Western region until recently, low genetic diversity could be attributed either to natural migration of European mink into Western Europe from Central Europe or to a putative human-mediated introduction of individuals from Central and/ or Eastern Europe. If the presence of the species in Western Europe is old enough, a rapid decline of the population in France for unknown reasons causing a severe genetic bottleneck followed by restoration of the population could also explain the observed genetic pattern. Alternatively, other more general but less obvious causes influencing also other species than the European mink could be underlying the evolutionary process. Unfortunately, the poor fossil record of European mink in the Western region and the uncertainty associated to estimated divergence times based on genetic data do not allow dating when exactly the species started inhabiting the region. Moreover, the rapid extinction process of European mink from Central Europe precludes any valid conclusions with regard to the feasibility of competing hypotheses. Future studies that may be capable of obtaining genetic data from museum specimens originated in Central Europe, as well as other focused in the population genetic structure of other related species (e.g. polecat, otter, stoat) could help discerning among the above-mentioned hypotheses.

### Conservation strategies

Since two-four isolated units of European mink exist, conservation criteria could be targeted at maintaining current genetic differences in these regions by managing them separately. However, our results indicate that throughout most of their evolutionary history, European mink populations were connected, and indeed likely formed a panmictic unit. Thus, the isolation of the units is viewed here as a recent human-induced event. The vast genetic differences detected among the distinct geographic areas are likely the result of the drastic population decline occurring during the past two centuries, although a founder effect cannot be precluded as an alternative source of the marked genetic divergence signature observed in W populations [10].

Hence, our results suggest that to return to the historical optimum of the species, European mink populations should actually be managed as a single management unit, and that strategies should be taken to promote gene flow among scattered existing populations. Actions to improve genetic connectivity are considered key to counteract inbreeding depression, as well as to preserve evolutionary potential and ensure long-term survival of endangered species [39–41]. Without such actions, the levels of genetic variability are currently so weak in some populations, that the risk of a rapid extinction is extremely high. To optimize conservation strategies, connectivity restoration programs need to minimize genetic threats arising from reduced outbreeding or loss of local adaptation [39, 42]. In addition, further *in-situ* actions (e.g., preserving an optimal ecological riverine niche, preventing infectious diseases, controlling American mink population) would minimize other factors increasing the extinction risk associated with the European mink [43–45].

## Conclusions

Based on both mt and nuclear (microsatellite) data, we found that the European mink populations of the Eastern European region (NE and SE) hold the highest genetic diversity. In contrast, the low values of genetic polymorphism and the structure detected in the W populations could be the result of founder events, possibly followed by genetic depauperation. Our results suggest that European mink populations were stable during Late Pleistocene climate oscillations, expanded through river connections after the last glaciation period, and later suffered relative high levels of extinction (e.g., in central Europe). Moreover, European mink likely experienced recent bottleneck events throughout Europe, consistent with the reported overall demographic decline of the species during the past centuries. According to the genetic patterns observed in this study, management strategies aimed to halter the ongoing decline of the endangered European mink should promote gene flow among scattered populations in order to improve genetic connectivity.

## Methods

### Sample collection

A total of 344 hair and tissue (skin, muscle or liver) samples were obtained from mink captured for tracking purposes, from specimens in museum collections and from traffic-killed individuals in France (*n* = 76), Spain (*n* = 94), Russia (*n* = 89), Estonia (*n* = 3), Belarus (*n* = 28), and Romania (*n* = 54). The performed research did not involve any experiment with animals and complies with international ethic guidelines. Samples were either stored in 96 % ethanol or frozen at −20 °C. For genetic analyses, each mtDNA and microsatellite data from all individuals were grouped according to i) their regional distribution (datasets I) into Northeastern Europe (NE; Russia, Belarus and Estonia), Southeastern Europe (SE; Romania), and Western Europe (W; France and Spain) or ii) their river drainage distribution (datasets II) into the Northern Dvina (Severnaya Dvina), Volga and Western Dvina (Zapadnaya Dvina) in Russia, Belarus and Estonia; the Danube in Romania; the Charentes, Garonne and Adour in France, and the Ebro and Cantabrian rivers in Spain (Fig. 1).

### DNA extraction and marker choice

DNA was extracted through standard proteinase K/ phenol-chloroform procedures [46] or using the QIAGEN® DNeasy Tissue Kit (Izasa, S. A., Barcelona).

#### mtDNA

A 614-bp fragment including the 3′-end of the cytochrome *b* gene, and the hypervariable region of the mt control region, was amplified using two PCR primers specifically designed for European mink according to the PCR conditions described in Cabria *et al*. [47]. Newly determined mtDNA sequences were deposited in GenBank under accession numbers EU548035–EU548051. Two mtDNA control region sequences of the polecat, *Mustela putorius*, retrieved from GenBank (AY962025 and AY962030 [48]) were used as outgroup taxa.

#### Microsatellites

A total of 11 polymorphic microsatellite loci were analyzed. Five specific loci (Mlut04, Mlut20, Mlut25, Mlut32 and Mlut35) were isolated directly from *Mustela lutreola* [12], whereas the rest were originally developed for *Mustela erminea* (Mer09, Mer22 and Mer41) and *Neovison vison* (Mvis22, Mvis72, Mvis75) [49]. The PCR conditions used are provided in Cabria *et al*. [12] and Cabria *et al*. [47]. Only individuals genotyped for nine or more loci were included in further genetic analyses. Microsatellites were amplified and genotyped anew from the samples of Michaux et al. (2005) to avoid potential cross calibration problems.

### Mitochondrial DNA analyses

#### Population diversity and demographic analyses

Sequences were aligned using default parameters of CLUSTALX v.2 [48] and further inspected by eye to maximize positional homology. Gapped positions were excluded from further analysis. Haplotype (H; [50]) and nucleotide (Π; [50]) diversity values were estimated both globally and separately for each population using Arlequin v.3.1 [51]. To detect departures (e.g., selection, demographic expansions or contractions) from the neutral model (random evolution), Tajima’s D [52], Fu’s Fs [53] and R_2_ [54] tests were performed using Arlequin v.3.1 and DNASP v.5 [55]. Statistical significance was tested by performing either 10,000 coalescent simulations in DNASP v.5 or 10,000 random permutations in Arlequin v.3.1. In addition, past population dynamic reconstructions were performed with Bayesian coalescent-based methods as implemented in BEAST v1.7.2 [56]. Bayesian skyline plots (BSPs) were generated using Markov chain Monte Carlo (MCMC) sampling to infer past changes in the effective population size (N_e_) of the Eastern (NE and SE) European mink populations (note that the W population has a single haplotype, which prevents further population genetic analyses). The models HKY + G [57] and F81 [58] were selected as best fit nucleotide substitution models for the NE and SE populations, respectively, using the Bayesian information criterion (BIC) as implemented in jModeltest v.0.1.1 [59, 60]. Simulations were run with a strict molecular clock using uniformly distributed priors. We used for clock calibration the divergence rate of 14.5 % Myr^−1^, which was estimated for the closely related *M. erminea* mtDNA complete cytochrome *b* and partial control region genes [61]. A piecewise-constant model of population size was selected with 10 groups. MCMC tests were run for 2 × 10^8^ iterations and sampled every 10,000 iterations with 10 % discarded as burn-in. To assess the robustness of parameter estimates, two independent runs were performed with identical settings and combined with LogCombiner [56]. Tracer v.1.5 [56] was used to visualize MCMC output and check for convergence and fluctuation of MCMC chains. The Bayesian skyline reconstructions were generated using Tracer to assess effective population size over time (N_e_τ), median estimate, and 95 % highest posterior density (HPD) limits. The generation time used to estimate the Ne was assumed to be one year [62].

#### Population structure analysis

Genetic structure among populations was determined by estimating pairwise differences between haplotypes. *Φ-statistics* [63] were estimated using Arlequin v.3.1, and their significance was determined with 90,000 permutation tests. The hierarchical distribution of mt genetic variation among populations was determined using an analysis of molecular variance (AMOVA) as implemented in Arlequin v.3.1. We examined how genetic variation was partitioned between the two major geographical areas (Western and Eastern Europe), as well as considering the NE and SE regions separately. Significances of Φ-statistics and of variance components were tested using 90,000 random permutations. In all multiple tests, *p*-values were adjusted using Bonferroni correction [64].

#### Phylogenetic and network analyses

All sequences were collapsed into distinct haplotypes using DNASP v.5 [55] and combined into a single data set. This data set was analyzed with three different methods of phylogenetic inference: Maximum Likelihood (ML; [58]) using PAUP v.4.0b10 [65], Bayesian inference (BI; [66]) using MrBayes v.3.2.0 [67] and coalescence-based methods using BEAST v.1.7 [56]. Best-fit models of nucleotide substitution were estimated using jModeltest based on Akaike information criteria (AIC) or BIC for the ML (TIM2 + I + G) and BI (HKY + G; [57] analyses, respectively. ML was performed using heuristic searches with 10 random addition and TBR branch swapping. BI was conducted as four simultaneous chains, each of 2 × 10^8^ generations, sampled every 1000 generations (10 % of trees were discarded as burn-in). Robustness was assessed through non-parametric bootstrap [68] proportions (BPs; 1000 pseudoreplicates) and Bayesian posterior probabilities (BPPs), respectively. Coalescence-based phylogenetic inference was performed using MCMC sampling. Model HKY + I + G [57] was selected as the best fit nucleotide substitution using the Akaike information criterion (AIC) as implemented in jModeltest v.0.1.1 [59, 60]. MCMCs were run for 2 × 10^8^ iterations and sampled every 10,000 iterations with 10 % discarded as burn-in using TreeAnnotator. Different models of coalescence were applied as the tree prior to investigate consistency in the results. MCMC analyses were run for 20 million iterations and sampled every 2000 iterations. The reconstructed trees had the same 50 % majority-rule consensus topology as shown in Fig. 2 (reconstructed using ML and BI; data not shown).

Intraspecific genetic variation was determined using two different networking approaches. First, mtDNA haplotypes were plotted on a median-joining (MJ) network applying NETWORK v.4.112 [46]. After network calculation, the MP option was applied for elimination of unnecessary median vectors [69]. We repeated the analyses varying the parameter epsilon (a weighted genetic distance measure), and no changes were observed in the reconstructed networks. A second haplotype network was constructed under parsimony with TCS v.1.21 [70].

### Microsatellite statistical analyses

#### Standard genetic variability analyses

Genetic diversity was estimated per locus and per population based on total number of alleles (N_A_), private alleles, allelic diversity (A), as well as observed (H_o_) and expected (H_e_) heterozygosities [71] using FSTAT v.2.93 [72] and Genetix v.4.05 [73]. Deviations from Hardy-Weinberg equilibrium (HWE) for each locus and across all loci, as well as genotypic linkage disequilibrium between all pairs of loci were tested in GenePop v.4 [74]. Statistical significance was tested by running a Monte Carlo Markov Chain (MCMC) consisting of 10,000 batches of 10,000 iterations each, with the first 10,000 iterations discarded before sampling [75]. *P*-values were adjusted with Bonferroni procedures that correct for the effect of multiple tests [64]. In addition, the unbiased Wright inbreeding coefficient F_IS_ [76] was used to define deviations from HWE.

The possible existence of null alleles and their frequencies were inferred using the EM algorithm [77] as implemented in FreeNA [78]. We also evaluated the impact of null alleles on the estimation of genetic differentiation. To estimate whether the analyzed microsatellite dataset provided sufficient statistical power to detect significant genetic differentiation, we used POWSIM [79]. Allele frequency homogeneity at each of the eleven loci, separately or combined, was assessed with Fisher’s exact and traditional chi-squared tests. Results indicated that the probability of detecting population structure was high, and statistically significant (data not shown). When F_ST_ was set to zero (which simulates no divergence among samples), the proportion of falsely significant values (α type I error) was in all cases lower than the intended value of 5 %.

#### Bayesian clustering analyses

Two different Bayesian clustering methods were used to determine the population structure of the European mink. First, we used STRUCTURE v2.2 [13]. The number of subpopulations (*k*) was calculated with no prior population information and an admixture model. We performed 10 series of independent runs for *K* from one to ten populations, setting default values with constant lambda (λ) and the same alpha (α) values for all populations. MCMC consisted of 10^5^ burn-in iterations followed by 10^6^ sampled iterations. Further, the modal value of lambda, *∆k* [14] was also calculated to infer the best value of *k*. Clusters were depicted using Distruct v.1.1 [80].

In addition, STRUCTURAMA v1.0 (http://fisher.berkeley.edu/structurama/) was used to verify congruence among results obtained with STRUCTURE, assuming that the number of populations is a random variable that follows a Dirichlet process prior [15]. The prior mean of the number of populations (α) was set as a random variable from 2 to 6, and 10^4^ MCMC cycles were run.

Finally, a spatial analysis of molecular variance, (SAMOVA 1.0, [81], was used to define partitions of sampling sites that are maximally differentiated from each other without any a priori assumption about population structure. This method identifies *k* genetically differentiated populations, where the proportion of total genetic variance (F_CT_) is maximized. The geographic coordinates for each region were calculated as the centre of the different localities. We tested a range of values of *k* from 2 to 5, using 100 simulated annealing steps.

#### Population differentiation

Genetic differentiation between populations was assessed based on two statistics: F_ST_ (infinite allele model, IAM; [76]), and R_ST_, (stepwise mutation model, SMM; [82]). The significance level was assessed by conducting 90,000 permutations, as implemented in the RstCalc package v.2.2 [83] and Arlequin v.3.1. An AMOVA test was performed to determine how genetic variation was partitioned within and among the same hierarchical scheme described above for mtDNA and the clusters inferred with STRUCTURE using Arlequin v.3.1. In all cases, *P*-values were adjusted using Bonferroni correction [64]. Patterns of genetic differentiation among all samples were visualized in a Factorial Correspondence Analysis (FCA) plot using Genetix v.4.05.

#### Biogeographic analyses

Approximate Bayesian Computation implemented in the DIYABC v.1.0.4 [84] was used to infer the colonization pathway of the European mink in Western Europe, as well as to assess how past/recent evolutionary history may have shaped the current genetic diversity and structure of the species, based on a comparison of a wide range of different colonization scenarios.

The biogeographic analyses were based on the microsatellite data set. We compared fourteen different biogeographic models assuming that NE and SE populations diverged from the same ancestral (source) population or from an admixture of two populations (Fig. 5). Because of constraints related to computational time, the space of all possible sequence of divergence could not be systematically explored. However, we tried to choose the most plausible scenarios on the basis of our knowledge of the European mink’s biology and historical information concerning this species. For historical model parametrization, these scenarios were described as a succession of events including population divergence and admixture. The first 6 scenarios are characterised by 3 steps of separation: step 1: separation between populations NE and SE; step 2: admixture between these populations; step 3: separation between populations FR and SP.

- Scenario 1 corresponds to a non-differentiation between the NE and SE populations at step 1, followed by an admixture at step 2. This leads to a new lineage corresponding to the FR population, which diverge at step 3 to give the SP population.
- Scenario 3 is similar to scenario 1 at the exception of step 2, which leads to a lineage corresponding the SP population. This will diverge at step 3 to give the FR population.
- Scenario 5 is also similar to scenario 1 but without differentiation between FR and SP populations at step 3.
- Scenario 2, 4, and 6 propose similar divergence as compared to the last three scenarios, respectively, at the exception of a differentiation between NE and SE populations at step 1.
- Scenarios from 7 to 14 are also characterized by three steps of divergence, but without admixture processes. Each of these scenarios is characterized by different possibilities of divergence leading to the four existing mink populations, from the most ancient lineages corresponding to the NE and SE populations to the most recent ones (SP and FR).

A total of 14 million simulations were run, providing one million simulations for each scenario. The parameters of each model (i.e. population sizes, timings of demographic events, mutation rates) were considered as random variables drawn from prior distributions (see Additional file 8).

The posterior probability of each scenario was estimated using a polychotomous logistic regression [84, 85] on the 1 % of simulated data sets closest to the observed data set, subject to a linear discriminant analysis as a pre processing step [86]. The selected scenario was that with the highest posterior probability value with a non-overlapping 95 % confidence interval.

### Availability of supporting data

mtDNA sequences: GenBank accession numbers, EU548035 (Mlh1), EU548036 (Mlh2), EU548037 (Mlh3), EU548038 (Mlh4), EU548039 (Mlh5), EU548040 (Mlh6), EU548041 (Mlh7), EU548042 (Mlh8), EU548043 (Mlh9), EU548044 (Mlh10), EU548045 (Mlh11), EU548046 (Mlh12), EU548047 (Mlh13), EU548048 (Mlh14), EU548049 (Mlh15), EU548050 (Mlh16), EU548051 (Mlh17), Data sets used in this study were deposited in TreeBase (http://purl.org/phylo/treebase/phylows/study/TB2:S17884).

## Additional files

Additional file 1:
**Estimated Φ**_**ST**_ **values for mtDNA.** Estimated Φ_ST_ values* for mtDNA obtained by comparing individuals grouped according to regional distribution (mtDNA dataset I) and drainage basin (mtDNA dataset II). Additional file 2:
**Analysis of molecular variance (AMOVA) based on mtDNA data.** *P* values in bold indicate a significant difference. Additional file 3:
**Estimates for F**_**ST**_ **and R**_**ST**_ **.** Estimates* for F_ST_ (below diagonal) and R_ST_ (above diagonal) between geographical region-pairs (microsatellite dataset I) and drainage basin pairs (microsatellite dataset II) from eleven microsatellite loci tested in European mink individuals. Values in bold indicate significance after sequential Bonferroni correction. Additional file 4:
**Analysis of molecular variance (AMOVA) based on microsatellite data.** Data represent eleven microsatellite loci. *P* values in bold indicate a significant difference. Additional file 5:
**Factorial correspondence analysis plot constructed by GENETIX using data for eleven microsatellite loci.** The plot depicts multivariate relationships among the European mink sampled. Each axis displays total variation percentages in allele frequencies. Additional file 6:
**Comparison of the posterior probabilities of all tested scenarios in the ABC analysis using a polychotomous Logistic regression approach.**
Additional file 7:
**Divergence time estimations for the main European mink’s evolutionary steps, obtained with diyABC analyses.**
Additional file 8:
**Model specification, prior distributions for demographic parameters and locus-specific mutation model parameters used for the diyABC analyses.** Type of parameters: (N) effective population size, (T) time of the event in generation, (A) admixture. Uniform distribution (UN) with 2 parameters: min and max; Gamma distribution (GA) with 2 parameters: mean and shape. The mutation model parameters for the microsatellite loci were the mean mutation rate (*μ*_mic_), the parameter determining the shape of the gamma distribution of individual loci mutation rate (*P*), and the Single Insertion Nucleotide rate (*SNI*).
