# Supplementary material for: Patterns of genetic variation in the endangered European mink (Mustela lutreola L., 1761)
Source: BMC Evol Biol. 2015 Jul 17;15:141. doi: 10.1186/s12862-015-0427-9 (PMC4504092; doi:10.1186/s12862-015-0427-9)
Supplement: Additional file 1: — Estimated Φ ST values for mtDNA. Estimated ΦST values* for mtDNA obtained by comparing individuals grouped according to regional distribution (mtDNA dataset I) and drainage basin (mtDNA dataset II). [file 12862_2015_427_MOESM1_ESM.doc]

**Additional file 1:** **Estimated Φ_ST_ values for mitochondrial DNA.**

| Estimated Φ_ST_ values^*^ for mitochondrial DNA obtained when individuals, grouped according to regional distribution (mtDNA dataset I) and drainage basins (mtDNA dataset II), were compared. | | | | | | | | | |
| --- | --- | --- | --- | --- | --- | --- | --- | --- | --- |
| mtDNA dataset I | Northeast | Southeast | West | mtDNA  dataset II | Northern Dvina | Volga | Western Dvina | Danube | Western rivers |
| Northeast | — |  |  | Northern Dvina | — |  |  |  |  |
|  |  |  |  | Volga | 0.071 | — |  |  |  |
|  |  |  |  | Western Dvina | 0.038 | **0.112** | — |  |  |
| Southeast | **0.586** | — |  | Danube | **0.677** | **0.696** | **0.572** | — |  |
| West | **0.622** | **0.879** | — | Western rivers | **0.789** | **0.817** | **0.662** | **0.879** | — |
| ^*^Bold values were significant after sequential Bonferroni corrections. | | | | | | | | | |
