# Supplementary material for: Patterns of genetic variation in the endangered European mink (Mustela lutreola L., 1761)
Source: BMC Evol Biol. 2015 Jul 17;15:141. doi: 10.1186/s12862-015-0427-9 (PMC4504092; doi:10.1186/s12862-015-0427-9)
Supplement: Additional file 2: — Analysis of molecular variance (AMOVA) based on mtDNA data. P values in bold indicate a significant difference. [file 12862_2015_427_MOESM2_ESM.doc]

**Additional file 2:** **Analysis of molecular variance (AMOVA) based on mitochondrial DNA data.**

| Analysis of molecular variance (AMOVA) of spatial genetic variation in European mink based on mitochondrial DNA data. Bold *P* values are significant values. | | | | | |
| --- | --- | --- | --- | --- | --- |
| Structure tested | Variance | | % variation | *F* *Statistics* | *P* |
| 1. One group (Russia, Belarus+Estonia^*^, Romania, France+Spain^**^) | | | | | |
| Among populations | | 1.004 | 63.89 |  |  |
| Within populations | | 0.568 | 36.11 | *F_ST_* = 0.638 | **<0.001** |
| 2. Two groups (Russia, Belarus+Estonia, Romania) vs. (France+Spain) | | | | | |
| Among groups | | 0.280 | 16.51 | *F_CT_ =* 0.165 | 0.495 |
| Among populations | | 0.847 | 50.00 | *F_SC_ =* 0.599 | **<0.001** |
| Within populations | | 0.568 | 33.49 | *F_ST_* = 0.665 | **<0.001** |
| 3. Two groups (Russia, Belarus+Estonia) vs. (Romania) | | | | | |
| Among groups | | 1.046 | 52.73 | *F_CT_ =* 0.527 | 0.334 |
| Among populations | | 0.155 | 7.83 | *F_SC_ =* 0.166 | **0.010** |
| Within populations | | 0.782 | 39.45 | *F_ST_* = 0.606 | **<0.001** |
| 4. One group (Northern Dvina+Pechora+Mezem, Volga+Caucasian, Western Dvina+Estonian, Danube, Charente, Garonne, Adour, Ebro and Cantabric rivers) | | | | | |
| Among populations | | 0.896 | 61.13 |  |  |
| Within populations | | 0.570 | 38.87 | *F_ST_* = 0.611 | **<0.001** |
| 5. Two groups (Northern Dvina+Pechora+Mezem, Volga+Caucasian, Western Dvina+Estonian, Danube rivers) vs. (Charente, Garonne, Adour, Ebro and Cantabric rivers) | | | | | |
| Among groups | | 0.461 | 27.18 | *F_CT_ =* 0.272 | 0.402 |
| Among populations | | 0.6647 | 39.21 | *F_SC_ =* 0.539 | **<0.001** |
| Within populations | | 0.5696 | 33.60 | *F_ST_* = 0.664 | **<0.001** |
| 6. Two groups (Northern Dvina+Pechora+Mezem, Volga+Caucasian, Western Dvina+Estonian rivers) vs. (Danube river) | | | | | |
| Among groups | | 1.112 | 56.42 | *F_CT_ =* 0.564 | 0.249 |
| Among populations | | 0.078 | 3.92 | *F_SC_ =* 0.090 | **0.002** |
| Within populations | | 0.787 | 39.66 | *F_ST_* = 0.603 | **<0.001** |
| Because of low number of samples collected, Belarus and Estonian^*^, as well as, French and Spanish^**^ individuals were pooled all together. | | | | | |
