# Supplementary material for: Patterns of genetic variation in the endangered European mink (Mustela lutreola L., 1761)
Source: BMC Evol Biol. 2015 Jul 17;15:141. doi: 10.1186/s12862-015-0427-9 (PMC4504092; doi:10.1186/s12862-015-0427-9)
Supplement: Additional file 3: — Estimates for F ST and R ST . Estimates* for FST (below diagonal) and RST (above diagonal) between geographical region-pairs (microsatellite dataset I) and drainage basin pairs (microsatellite dataset II) from eleven microsatellite loci tested in European mink individuals. Values in bold indicate significance after sequential Bonferroni correction. [file 12862_2015_427_MOESM3_ESM.doc]

**Additional file 3: Estimates for F_ST_ and R_ST_.**

| Estimates^*^ for F*_ST_* (below diagonal) and R_ST_ (above diagonal) between geographical regions-pairs (microsatellite dataset I) and drainage basins sample pairs (microsatellite dataset II) from eleven microsatellite loci tested in European mink individuals. Bold values were significant after sequential Bonferroni corrections. | | | | | | | | | | | | | |
| --- | --- | --- | --- | --- | --- | --- | --- | --- | --- | --- | --- | --- | --- |
| Microsatellite dataset I | Northeast | Southeast | West | Microsatellite dataset II | North Dvina | West Dvina | Volga | Danube | Charentes | Garonne | Adour | Cantabrian | Ebro |
| Northeast | — | **0.073** | **0.067** | North Dvina | — | **0.064** | 0.004 | **0.070** | **0.109** | **0.098** | **0.077** | **0.101** | **0.117** |
|  |  |  |  | West Dvina | **0.021** | — | **0.033** | **0.120** | **0.136** | **0.134** | **0.154** | **0.169** | **0.190** |
|  |  |  |  | Volga | **0.022** | **0.041** | — | **0.078** | **0.103** | **0.098** | **0.065** | **0.089** | **0.099** |
| Southeast | **0.128** | — | **0.093** | Danube | **0.130** | **0.137** | **0.160** | — | **0.232** | **0.199** | **0.155** | **0.109** | **0.101** |
| West | **0.184** | **0.282** | — | Charentes | **0.174** | **0.175** | **0.215** | **0.320** | — | 0.006 | **0.082** | **0.212** | **0.264** |
|  |  |  |  | Garonne | **0.170** | **0.170** | **0.215** | **0.304** | 0.004 | — | **0.052** | **0.184** | **0.235** |
|  |  |  |  | Adour | **0.168** | **0.172** | **0.198** | **0.281** | **0.075** | **0.047** | — | **0.117** | **0.104** |
|  |  |  |  | Cantabric | **0.192** | **0.186** | **0.236** | **0.243** | **0.274** | **0.217** | **0.118** | — | **0.063** |
|  |  |  |  | Ebro | **0.307** | **0.304** | **0.330** | **0.366** | **0.359** | **0.299** | **0.177** | **0.112** | — |
