# Supplementary material for: Patterns of genetic variation in the endangered European mink (Mustela lutreola L., 1761)
Source: BMC Evol Biol. 2015 Jul 17;15:141. doi: 10.1186/s12862-015-0427-9 (PMC4504092; doi:10.1186/s12862-015-0427-9)
Supplement: Additional file 4: — Analysis of molecular variance (AMOVA) based on microsatellite data. Data represent eleven microsatellite loci. P values in bold indicate a significant difference. [file 12862_2015_427_MOESM4_ESM.doc]

**Additional file 4: Analysis of molecular variance (AMOVA) based on microsatellites data.**

| Analysis of molecular variance (AMOVA) of spatial genetic variation in European mink based on eleven microsatellites data. Bold *P* values are significant values. | | | | | | |
| --- | --- | --- | --- | --- | --- | --- |
| Structure tested | Variance | | | % total | *F* *Statistics* | *P* |
| 1. One group (Russia, Belarus+Estonia, Romania, France, Spain) | | | | | | |
| Among populations | | | 0.654 | 22.44 |  |  |
| Within populations | | | 2.259 | 77.56 | *F_ST_* = 0.224 | **<0.001** |
| 2. Two groups (Russia, Belarus+Estonia, Romania) vs. (France, Spain) | | | | | | |
| Among groups | | | 0.335 | 11.07 | *F_CT_ =* 0.111 | 0.101 |
| Among populations | | | 0.434 | 14.33 | *F_SC_ =* 0.161 | **<0.001** |
| Within populations | | | 2.259 | 74.60 | *F_ST_* = 0.254 | **<0.001** |
| 3. Two groups (Russia, Belarus+Estonia) vs. (Romania) | | | | | | |
| Among groups | | | 0.354 | 11.02 | *F_CT_ =* 0.111 | 0.332 |
| Among populations | | | 0.068 | 2.13 | *F_SC_ =* 0.161 | **0.001** |
| Within populations | | | 2.788 | 86.86 | *F_ST_* = 0.131 | **<0.001** |
| 4. Four groups (Russia, Belarus+Estonia) vs. (Romania) vs. (France) vs. (Spain) | | | | | | |
| Among groups | | | 0.604 | 20.54 | *F_CT_ =* 0.205 | 0.101 |
| Among populations | | | 0.077 | 2.61 | *F_SC_ =* 0.329 | **<0.001** |
| Within populations | | | 2.259 | 76.85 | *F_ST_* = 0.232 | **<0.001** |
| 5. One group (Northern Dvina+Pechora+Mezem, Volga+Caucasian, Western Dvina+Estonian, Danube, Charente, Garonne, Adour, Ebro and Cantabric rivers) | | | | | | |
| Among populations | | 0.615 | | 21.63 |  |  |
| Within populations | | 2.228 | | 78.37 | *F_ST_* = 0.216 | **<0.001** |
| 6. Two groups (Northern Dvina+Pechora+Mezem, Volga+Caucasian, Western Dvina+Estonian, Danube rivers) vs. (Charente, Garonne, Adour, Ebro and Cantabric rivers) | | | | | | |
| Among groups | | 0.431 | | 14.24 | *F_CT_ =* 0.143 | 0.008 |
| Among populations | | 0.365 | | 12.07 | *F_SC_ =* 0.141 | **<0.001** |
| Within populations | | 2.228 | | 73.68 | *F_ST_* = 0.263 | **<0.001** |
| 7. Two groups (Northern Dvina+Pechora+Mezem, Volga+Caucasian, Western Dvina+Estonian rivers) vs. (Danube river) | | | | | | |
| Among groups | | 0.356 | | 11.11 | *F_CT_ =* 0.111 | 0.250 |
| Among populations | | 0.084 | | 2.61 | *F_SC_ =* 0.029 | **<0.001** |
| Within populations | | 2.763 | | 86.27 | *F_ST_* = 0.137 | **<0.001** |
| 8. Four groups (Northern Dvina+Pechora+Mezem, Volga+Caucasian, Western Dvina+Estonian rivers) vs. (Danube river) vs. (Charente, Garonne, Adour rivers) vs. (Ebro and Cantabric rivers) | | | | | | |
| Among groups | | 0.6124 | | 20.86 | *F_CT_ =* 0.209 | **<0.001** |
| Among populations | | 0.096 | | 3.27 | *F_SC_ =* 0.041 | **<0.001** |
| Within populations | | 2.227 | | 75.87 | *F_ST_* = 0.241 | **<0.001** |
