# Supplementary material for: Patterns of genetic variation in the endangered European mink (Mustela lutreola L., 1761)
Source: BMC Evol Biol. 2015 Jul 17;15:141. doi: 10.1186/s12862-015-0427-9 (PMC4504092; doi:10.1186/s12862-015-0427-9)
Supplement: Additional file 5: — Factorial correspondence analysis plot constructed by GENETIX using data for eleven microsatellite loci. The plot depicts multivariate relationships among the European mink sampled. Each axis displays total variation percentages in allele frequencies. [file 12862_2015_427_MOESM5_ESM.doc]

**Additional file 5: Factorial correspondence analysis plot performed by GENETIX with eleven microsatellites.** FCA shows the multivariate relationships of European minks sampled. Each axe displays the percentage of the total variation in allele frequencies.
