# Supplementary material for: Patterns of genetic variation in the endangered European mink (Mustela lutreola L., 1761)
Source: BMC Evol Biol. 2015 Jul 17;15:141. doi: 10.1186/s12862-015-0427-9 (PMC4504092; doi:10.1186/s12862-015-0427-9)
Supplement: Additional file 6: — Comparison of the posterior probabilities of all tested scenarios in the ABC analysis using a polychotomous Logistic regression approach. [file 12862_2015_427_MOESM6_ESM.doc]

**Additional file 6: Comparison of the posterior probabilities of all tested scenarios in the ABC analysis using a polychotomous Logistic regression approach.**

**
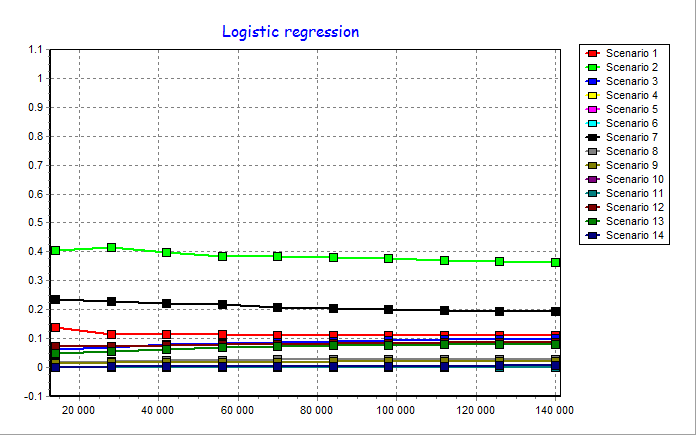
**
