# Supplementary material for: Patterns of genetic variation in the endangered European mink (Mustela lutreola L., 1761)
Source: BMC Evol Biol. 2015 Jul 17;15:141. doi: 10.1186/s12862-015-0427-9 (PMC4504092; doi:10.1186/s12862-015-0427-9)
Supplement: Additional file 7: — Divergence time estimations for the main European mink’s evolutionary steps, obtained with diyABC analyses. [file 12862_2015_427_MOESM7_ESM.doc]

**Additional file 7: Divergence time estimations for the main European mink’s evolutionary steps, obtained with diyABC analyses.**
