# Supplementary material for: Patterns of genetic variation in the endangered European mink (Mustela lutreola L., 1761)
Source: BMC Evol Biol. 2015 Jul 17;15:141. doi: 10.1186/s12862-015-0427-9 (PMC4504092; doi:10.1186/s12862-015-0427-9)
Supplement: Additional file 8: — Model specification, prior distributions for demographic parameters and locus-specific mutation model parameters used for the diyABC analyses. Type of parameters: (N) effective population size, (T) time of the event in generation, (A) admixture. Uniform distribution (UN) with 2 parameters: min and max; Gamma distribution (GA) with 2 parameters: mean and shape. The mutation model parameters for the microsatellite loci were the mean mutation rate (μ mic), the parameter determining the shape of the gamma distribution of individual loci mutation rate (P), and the Single Insertion Nucleotide rate (SNI). [file 12862_2015_427_MOESM8_ESM.doc]

**Additional file 8:** **Model specification, prior distributions for demographic parameters and locus-specific mutation model parameters used for the diyABC analyses.** Type of parameters: (N) effective population size, (T) time of the event in generation, (A) admixture. Uniform distribution (UN) with 2 parameters: min and max; Gamma distribution (GA) with 2 parameters: mean and shape. The mutation model parameters for the microsatellite loci were the mean mutation rate (*µ*_mic_), the parameter determining the shape of the gamma distribution of individual loci mutation rate (*P*), and the Single Insertion Nucleotide rate (*SNI*).
